# A randomised controlled trial of an intervention to promote early presentation of breast cancer in older women: effect on breast cancer awareness

**DOI:** 10.1038/sj.bjc.6605389

**Published:** 2009-12-03

**Authors:** L Linsell, L J L Forbes, M Kapari, C Burgess, L Omar, L Tucker, A J Ramirez

**Affiliations:** 1 King's College London, Cancer Research UK Promoting Early Presentation Group, Institute of Psychiatry, St Thomas' Hospital, London SE1 7EH, UK

**Keywords:** aged, awareness, breast neoplasms, complex intervention, delayed presentation, randomised controlled trials

## Abstract

**Background:**

There is virtually no evidence for the effectiveness of interventions to promote early presentation in breast cancer. We aimed to test the efficacy of an intervention to equip older women with the knowledge, skills, confidence and motivation to detect symptoms and seek help promptly, with the aim of promoting early presentation with breast cancer symptoms.

**Methods:**

We randomised 867 women aged 67–70 years attending for their final routine appointment on the UK NHS Breast Screening Programme to receive: a scripted 10-min interaction with a radiographer plus a booklet, a booklet alone or usual care. The primary outcome was whether or not a woman was breast cancer aware based on knowledge of breast cancer symptoms and age-related risk, and reported breast checking.

**Results:**

At 1 month, the intervention increased the proportion who were breast cancer aware compared with usual care (interaction arm: 32.8% *vs* 4.1%; odds ratio (OR): 24.0, 95% confidence interval (CI): 7.7–73.7; booklet arm: 12.7% *vs* 4.1%; OR: 4.4, 95% CI: 1.6–12.0). At 1 year, the effects of the interaction plus booklet, and the booklet, on breast cancer awareness were largely sustained, although the interaction plus booklet remained much more effective.

**Conclusions:**

An intervention to equip older women with the knowledge, skills, confidence and motivation to detect breast cancer symptoms and seek help promptly increases breast cancer awareness at 1 year. Future research will evaluate whether the intervention promotes early presentation and reduces breast cancer mortality.

Women in the United Kingdom have poorer survival from breast cancer than many other Western European countries (Berrino *et al*, 2007), and differences in stage at diagnosis are largely responsible (Sant *et al*, 2003). Late stage at diagnosis is almost certainly due to late presentation by some women and delays in onward referral by some general practitioners. In the developed world, 17–35% of women with breast cancer delay presenting for >3 months, and 9–20% delay >6 months (Westcombe *et al*, 1999; Arndt, 2002). We have found no high quality evidence of effectiveness of interventions to promote early presentation in breast, or any other cancer (Austoker *et al*, 2009).

Risk factors for delay in presentation in breast cancer include older age, less education and presenting with non-lump symptoms (Ramirez *et al*, 1999). Older women have particularly poor knowledge of non-lump symptoms and the increase in breast cancer risk with age (Grunfeld *et al*, 2002; Linsell *et al*, 2008). About 20% of older women report that they never look at or feel their breasts (Linsell *et al*, 2008). We hypothesise that breast cancer awareness, which might include breast cancer knowledge and the confidence, skills and motivation to detect symptoms and seek help appropriately, will reduce delays in presentation.

We have built two versions of an intervention to promote early presentation of breast cancer in older women (Burgess *et al*, 2008) aiming to equip them with breast cancer awareness: a booklet containing health-promoting messages and a scripted one-to-one interaction with a radiographer, supported by the booklet, both designed to be delivered during the final routine appointment on the NHS Breast Screening Programme, a setting giving access to most English women aged 67–70 years. We targeted older women because they are at higher risk of breast cancer (Cancer Research UK, 2008), are more likely to delay presentation (Ramirez *et al*, 1999) and have poorer survival (Office for National Statistics, 2008). The positive predictive value of breast symptoms for breast cancer is higher in older than younger women (Nichols *et al*, 1981). We based the design of the intervention on a theoretical framework for delayed presentation (Bish *et al*, 2005), and incorporated techniques to maximise the probability of behaviour change (Rollnick and Miller, 1995; Gollwitzer, 1999; Jepson, 2000; Wardle *et al*, 2003).

We have developed and evaluated the intervention in line with the Medical Research Council guidance on complex interventions (Medical Research Council, 2008). We have previously shown in a before-and-after exploratory trial that the intervention increased breast cancer awareness in older women at 6 months (Burgess *et al*, 2009). We report here the 1 month and 1 year results of a randomised controlled trial (RCT) of efficacy of the 10-min interaction with a radiographer supported by a booklet *vs* the booklet alone *vs* usual care alone.

## Materials and methods

### Participants and setting

Participants were a consecutive series of women aged 67–70 attending final routine appointments on the NHS Breast Screening Programme. All women attending were eligible, unless they had a significant disorder that may have affected their ability to consent or participate, insufficient English or other language difficulties, or were going overseas during the subsequent 6 months. The women were recruited from seven breast screening units in London and Surrey.

### Procedure

The screening units sent an invitation letter and information sheet to each potential participant 2 weeks before her appointment. On the day of attendance, a trained radiographer assessed whether the woman was eligible and obtained written informed consent. After completing a baseline questionnaire, women were randomly allocated to: usual care, booklet alone or the 10-min one-to-one interaction supported by the booklet, in addition to usual care. We sent trial-specific postal questionnaires at 1 and 12 months after randomisation to collect outcome data.

### The intervention

#### Usual care

The screening unit receptionist informed each woman who had received her final routine mammogram that she was no longer eligible for routine screening, advised her that she might continue to be screened every 3 years on request, and provided a card with contact details and a suggested date for contact.

#### The booklet

In addition to usual care, a radiographer gave a booklet to each woman who had received her final routine mammogram. The booklet conveyed key breast cancer awareness messages, including:

- A list of breast cancer symptoms;
- Age-related and absolute risk of developing breast cancer;
- How to detect a breast change;
- What to do on discovering a breast change;
- A strong direct recommendation to seek medical attention immediately on discovering a breast change, outlining the benefits of prompt help seeking and suggestions for overcoming barriers such as embarrassment and fear;
- A direct recommendation to tell someone close in the event of discovering a breast change;
- An action plan to be completed by the woman about how she will be breast aware and what she will do on discovering a breast change;
- A series of statements describing possible positive feelings (relief, reassurance, satisfaction), resulting from seeking help immediately with a breast change;
- A reminder that she might request further breast screening.

#### The interaction plus booklet

In addition to usual care, women received a scripted 10-min one-to-one interaction with a radiographer or research psychologist. During this, the radiographer/psychologist verbally delivered all the messages in the booklet in a positive, collaborative and motivational style, referred to the booklet throughout and gave it to the woman to take home. She also showed photographs of breast cancer signs and demonstrated and rehearsed breast checking using a silicone breast. The radiographer was able to tailor the key messages by checking the woman's understanding and answering any questions.

#### Quality assurance and quality control for the interaction plus booklet

After receiving training and being assessed as competent, five radiographers and two research psychologists delivered the interactions, and received ongoing performance feedback throughout. All interactions were video recorded and quality of each was assessed by an independent rater using a trial-specific quality checklist assessing content and style of delivery. Quality scores were standardised on a scale of 0–100.

### Outcomes

We measured outcomes at baseline and 1, 6 and 12 months after randomisation using a trial-specific questionnaire, an earlier version of which we have used in a survey (Linsell *et al*, 2008) and an exploratory trial (Burgess *et al*, 2009). We have demonstrated good test–retest reliability (Linsell, 2006) and sensitivity to change (Burgess *et al*, 2009).

The primary outcome was proportion of women achieving breast cancer awareness at 1 month, measured using a breast cancer awareness score. The score was a composite of responses to three questions from the questionnaire, relating to knowledge of symptoms, knowledge of age-related risk and reported breast checking:

*Knowledge of breast cancer symptoms*: ‘*Do you know any of the warning signs of breast cancer? If yes, please circle the signs you know below*’. Women circled symptoms on a scattered list of 11 symptoms (two lump and nine non-lump). To score one point, the woman had to identify at least five non-lump symptoms, that is, over half;

*Knowledge of age-related risk*: ‘*In the next year who is most likely to get breast cancer?*’ Response categories: a 30-year-old woman, a 50-year-old woman, a 70-year-old woman, a woman of any age. To score one point, the woman had to identify that a 70-year-old woman was most likely to get breast cancer;

*Breast checking*: ‘*How often do you check your breasts?*’ Response categories: rarely or never, at least every 6 months, at least once a month, at least once a week. To score one point, the woman had to report checking her breasts at least once a month or at least once a week.

Each item was given equal weighting and contributed one point to the total score (range: 0–3). To achieve breast cancer awareness, the woman had to respond correctly to all three items.

We also collected data on relationship status, education and ethnicity at baseline. To estimate socio-economic status, we used the Index of Multiple Deprivation (Communities and Local Government, 2007) based on area of residence. This is a measure of deprivation at the small area level (32 482 areas in England) based on seven dimensions: income, employment, health, education, housing and services, living environment and crime. Every area in England is ranked from 1 (most deprived) to 32 482 (least deprived); median rank is 16 241. We assigned each woman a rank of Index of Multiple Deprivation according to the rank of her area of residence.

### Sample size

We estimated that 2% of women would be breast cancer aware at baseline (Burgess *et al*, 2009) and that there would be a 12% difference between trial arms. Incorporating a design effect to take account of clustering by centre and radiographer (Lee and Thompson, 2005) (assuming an intracluster correlation coefficient of 0.08 and 14 participants per centre–radiographer cluster) and allowing for 70% response, we required 238 women per arm (total 714) with a significance level of 5% (two-sided) and power of 80%.

### Randomisation

We randomised women individually on the day of attendance, with equal probability of assignment to each arm. The trial statistician computer generated the allocation sequence using stratified block randomisation with centre and radiographer as stratification variables (block sizes of three, six and nine). To ensure concealment, assignments were enclosed in sequentially numbered, opaque, sealed envelopes and stored by the trial coordinator before randomisation. The radiographer recorded each participant's trial identification number on the envelope before opening it.

### Statistical analysis

We analysed the data by intention to treat: all participants were analysed in the groups to which they were allocated. We summarised continuous variables using means, standard deviations, medians and ranges, and categorical variables using counts and percentages. We used two-sided significance tests, taking *P*=0.05 as significant. All analyses were performed using Stata version 10.0. The primary comparative analyses for all outcomes examined the difference between baseline and 1, 6 and 12 months for each pair of intervention groups. We used robust generalised estimating equations (Zeger and Liang, 1986) with unstructured correlation structure using a logit link and binomial distribution for the outcomes. This method takes account of the correlation between repeated observations from the same individual. To examine the intervention effect, we tested the interaction between intervention group and time in each model, and presented odds ratios (OR) with 95% confidence intervals (CIs). We also analysed the data adjusting for stratification variables, relationship status, education, ethnicity and Index of Multiple Deprivation, fitting categorical variables as binary variables and Index of Multiple Deprivation on a continuous scale (0–100). We calculated the intracluster correlation coefficient for radiographer and centre using one-way analysis of variance, adjusting for unequal cluster size (Fleiss, 1981; Armitage and Berry, 1994).

## Results

### Flow of participants

**Figure 1:**
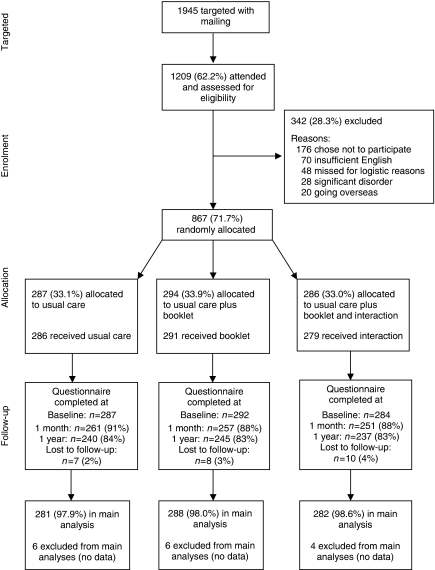
Flow of participants through trial.

Between August 2007 and May 2008, we randomised 867 women to one of three arms: usual care (*n*=287), the booklet in addition to usual care (*n*=294) and the interaction supported by the booklet in addition to usual care (*n*=286) (Figure 1). Only 15% (176 out of 1209) of women who were assessed for eligibility chose not to participate. Eleven women did not receive the allocated intervention and 25 were lost to follow-up (20 withdrew consent, three moved with no forwarding address, one for medical reasons and one died). We included women with data for the primary outcome on at least one occasion in the main analysis (*n*=851). We were unable to measure any outcomes for four women who did not complete any questionnaires (two booklet arm, two interaction arm). We received breast cancer awareness questionnaires from 89% of those randomised at 1 month and 83% at 1 year; response rates were similar in each arm (Figure 1).

### Baseline socio-demographic characteristics

Socio-demographic characteristics were well balanced across the arms (Table 1), except for a slight difference in the proportion of women with no educational qualifications (35% usual care, 41% booklet and 44% interaction arm). Women in the booklet arm had a similar deprivation score to the English median, but women in the usual care and interaction plus booklet arms lived in somewhat more deprived areas. For women invited for screening but not recruited, the only characteristic we had data was postcode (and, therefore, rank of Index of Multiple Deprivation). The median rank for the 1078 women not recruited was 9068 (interquartile range (IQR): 4784–15 802) compared with 15 664 (IQR: 8589–25 989) for the 867 women in the trial, so women who did not take part lived in more deprived areas than those who did.

### Breast cancer awareness

**Figure 2:**
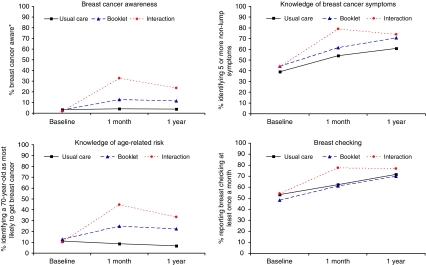
Breast cancer awareness and component items at baseline, 1 month and 1-year after randomisation. ^*^A woman scored three points on the breast cancer awareness score if she: identified at least five non-lump symptoms (one point), identified that a 70-year-old woman is most at risk of breast cancer (one point) and reported checking her breasts at least once a month (one point).

Table 2 shows the main results for breast cancer awareness score and its components for baseline, 1 month and 1 year (6 months data are not presented). Figure 2 illustrates breast cancer awareness and components of the score over the 12-month period. Overall, only 2.7% of women were breast cancer aware at baseline. At 1 month, the interaction plus booklet increased the proportion who were breast cancer aware compared with usual care (32.8% *vs* 4.1%; OR: 24.0, 95% CI: 7.7–73.7), as did the booklet, although the effect of the booklet was much less striking (12.7% *vs* 4.1%; OR: 4.4, 95% CI: 1.6–12.0). At 1 year, the effect of the interaction plus booklet, and the booklet alone, on breast cancer awareness remained significant, with the interaction plus booklet remaining more effective. The results of the adjusted analysis were similar, although estimated differences between arms were mostly slightly larger (data not shown).

### Knowledge of breast cancer symptoms

**Figure 3:**
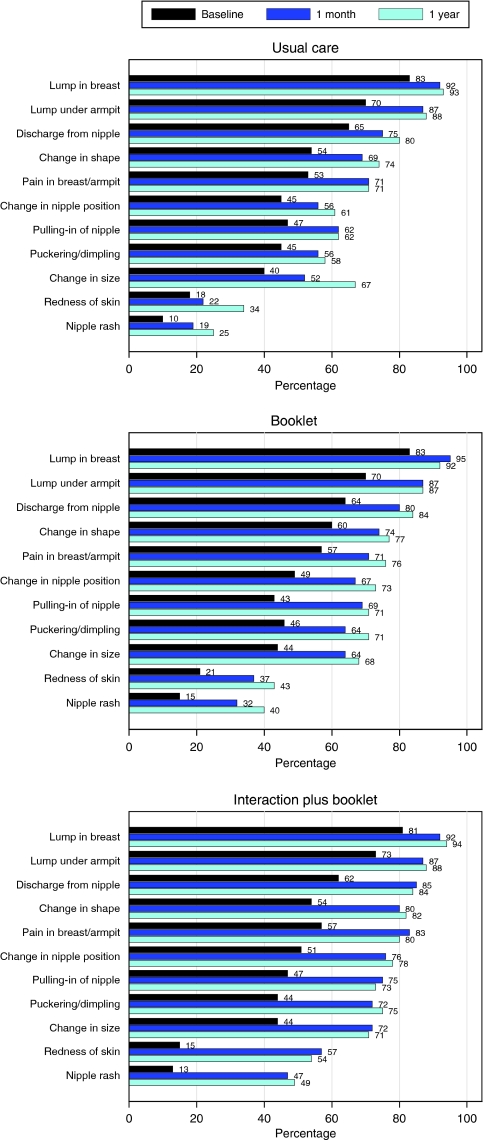
Proportions of women identifying symptoms of breast cancer at baseline, 1 month and 1-year after randomisation.

Forty-two per cent of women were able to identify five or more non-lump symptoms at baseline. At 1 month, the interaction plus booklet increased the proportion of women able to identify five or more non-lump symptoms compared with usual care (78.9% *vs* 54.2%; difference 24.7%; OR: 2.5, 95% CI: 1.7–3.6) but the booklet alone did not (61.6% *vs* 54.2%; difference 7.4%; OR: 1.1, 95% CI: 0.8–1.5). The increase in knowledge of symptoms associated with the interaction plus booklet was maintained at 1 year. Before receiving the intervention, the women were able to identify a median of four non-lump symptoms from the list of nine (IQR: 2–6), and most recognised a lump in the breast or armpit as symptoms. At 1 year, this increased to a median of six symptoms (IQR: 4–9) among those receiving the booklet only, and to seven (IQR: 4–9) among those receiving the interaction plus booklet. The intervention had most impact on the two least recognised symptoms, redness of skin and nipple rash (Figure 3).

### Knowledge of age-related risk

Only 11.4% of the women knew that a 70-year-old woman was most at risk of breast cancer at baseline. At 1 month, the interaction plus booklet increased the proportion knowing that a 70-year-old woman was at most risk of breast cancer compared with usual care (44.7% *vs* 8.7%; difference 36.0%: OR: 9.5, 95% CI: 5.1–17.6), as did the booklet alone (24.9% *vs* 8.7%; difference 16.2%; OR: 3.2, 95% CI: 1.8–5.8). At 1 year, the effect of the interaction plus booklet and the booklet alone remained significant.

### Breast checking

About half of the women reported checking their breasts at least once a month at baseline. At 1 month, the interaction plus booklet increased the proportion of women checking their breasts at least monthly compared with usual care (77.7% *vs* 62.5%; difference 15.2%; OR: 2.0, 95% CI: 1.4–2.8), but the booklet alone did not (61.3% *vs* 62.5%; difference −1.2%; OR: 1.2, 95% CI: 0.9–1.6). The effect of the interaction plus booklet was no longer significant at 1 year.

### Intervention delivery

A total of 279 interactions were conducted and 82% were video recorded and assessed by an independent rater (38 were not usable due to technical faults, five were incomplete, four were audio taped only, two women refused recording and one tape was lost). Quality of intervention delivery was high: median content score was 96 (range: 68–100) (only five interactions (2%) scored <80); median style score was 85 (range: 37–100) (79 interactions (35%) scored <80). One per cent of the total variability in the breast cancer awareness score at 1 month was attributable to radiographer, and 0.6% to centre.

## Discussion

The intervention increased breast cancer awareness among older women compared with usual care at 1 month, with the interaction supplemented by a booklet having a greater effect than the booklet alone. Thirty-three per cent of those receiving the interaction plus booklet and 13% of those receiving the booklet alone were breast cancer aware compared with 4% of women receiving usual care. These improvements in breast cancer awareness were sustained at 12 months although were somewhat less marked (24% of the interaction plus booklet group and 12% of the booklet only group compared with 4% of the usual care group). Of the three components of breast cancer awareness, the interaction plus booklet and the booklet alone had the most marked effect on knowledge of age-related risk. The interaction plus booklet was also associated with a statistically significant increase in knowledge of breast cancer symptoms.

Only 3% of the women were breast cancer aware (as defined prospectively for this study) at baseline. This may explain why so many women delay presenting with breast cancer symptoms and have poor survival as a result. Knowledge of age-related risk was particularly poor (only 11% were aware that a 70-year-old woman was at higher risk than a 30-year-old woman or a 50-year-old woman), perhaps because of heavy media coverage of younger women with breast cancer and the current upper age limits on the NHS Breast Screening Programme.

The efficacy of the booklet alone was limited. It was important to test the booklet alone, as it would be cheaper to deliver on the NHS than the one-to-one interaction. The interaction plus booklet was probably more effective because it incorporated features thought to promote behaviour change: a direct recommendation from a health professional (Jepson, 2000), tailoring (Jepson, 2000) and positive motivational style and verbal persuasion (Rollnick and Miller, 1995; Wardle *et al*, 2003).

Knowledge of non-lump symptoms and reported breast checking increased quite markedly in the women who received usual care alone over the 12-month follow-up. This may be due to what has been called the ‘mere measurement’ effect (Godin *et al*, 2008): either the questionnaire itself increased awareness, or women started to guess the correct, or most appropriate, answers because they were repeatedly asked the same question.

A systematic review of interventions to promote cancer awareness found very limited evidence of effectiveness of any interventions. It found only five RCTs of interventions to promote cancer awareness aimed at individuals, of moderate to good quality (Austoker *et al*, 2009). All found more modest effects on cancer awareness than we achieved. The trial finding the greatest effect was of an intensive intervention (tailored written information with a reinforcing newsletter at 12 months plus two telephone counselling sessions) primarily aiming to increase breast screening uptake. It increased the proportion who gave the correct answer to a question about age-related risk by 12% compared with usual care after 2 years (Rimer *et al*, 2002). In our study, the interaction plus booklet, which increased the proportion correctly identifying a 70-year-old woman as at higher risk than a 50-year-old woman or 30-year-old woman by 27% at 12 months, compares well with this. Less intensive interventions such as mailed information (de Nooijer *et al*, 2004) and interactive computer programmes (Glazebrook *et al*, 2006) increased cancer awareness more modestly. The effects of the interaction plus booklet on reported breast checking at least monthly (77% *vs* 72% after 12 months) are similar to those found in trials to promote breast self-examination: a RCT in the United States found that a 45-min class increased the proportion who reported monthly breast self-examination from 51% to 62% after 6 months (Strickland *et al*, 1997).

Our intervention is not a tutorial in breast self-examination. The evidence to support systematic, regular breast self-examination is weak: a Cochrane systematic review found that breast self-examination did not reduce mortality and increased investigations (Kosters and Gotzsche, 2003). However, both trials included in the review recruited women under the age of 67 years; whether breast checking, or even breast self-examination, would increase detection rates and reduce mortality in older women is unknown. What seems highly unlikely is that women who never look at or touch their breasts (20% of older women (Linsell *et al*, 2008)) will detect symptoms early; our intervention is designed to encourage simply looking and touching.

Strengths of our trial were the high level of participation (84% of eligible women were randomised) and the high response to follow-up (83% at 12 months). The usual care arm had slightly higher levels of education than the interaction arm; however, adjusting for baseline characteristics did not significantly change the size of the estimates.

The NHS Breast Screening Programme is an efficient setting for recruiting large numbers of healthy older women: uptake of breast screening in women aged 65–70 years is over 70% (NHS Breast Screening Programme, 2009). Women who take up breast screening live in less deprived areas than those who do not (Banks *et al*, 2002; Maheswaran *et al*, 2006), although the women in our study lived in slightly more deprived areas than the English average. Should the intervention be implemented across more affluent populations, it is likely that women receiving it would be of higher socio-economic status than those not receiving it. However, these women are at higher risk of breast cancer (Threlfall and Woodman, 2001; Shack *et al*, 2008) so it is appropriate to target them in this setting.

We developed a score for measuring breast cancer awareness to be used in surveys and trials. Currently, there is no universally accepted measure of breast cancer awareness (although the breast cancer module of the Cancer Awareness Measure is being developed) and no published agreement on what the concept means. We argue that it is not a single construct, so, in developing our measure, we included three constructs that we felt encompass the minimum information women need to be able to present promptly with breast cancer symptoms: why to look for them (magnitude of risk), what to look for (the range of symptoms) and how to look for them (to look at and feel their breasts).

Health professional-delivered complex interventions such as ours are prone to variability in the quality of delivery because they are made up of many components and are operator dependent, and this may influence whether they work or not. In psychotherapy trials, a significant amount of the variability in participant outcome has been shown to be attributable to variable delivery by different therapists (Crits-Christoph *et al*, 1991; Okiishi *et al*, 2003; Wampold and Brown, 2005). In our trial, we found no evidence of important variation in the quality of delivery of the interaction between those delivering it.

We recognise that some of the symptoms included in the intervention and the questionnaire (e.g. lump in armpit) are likely to indicate disease of a worse prognosis. We included these symptoms because even in women with more advanced disease than stage I, earlier detection may improve prognosis. In addition, we felt it important to include all symptoms of breast cancer in the list as their absence might give a misleading message.

It could be argued that inviting older women for screening on the NHS Breast Screening Programme might be a better way of promoting earlier diagnosis in breast cancer (currently the upper age limit for routine invitation is 70; this is soon to increase to 73). However, breast screening is expensive and the cost-effectiveness of inviting older women for screening is not clear. Raising awareness is likely to compare favourably in cost-effectiveness terms, and may have a more prolonged effect than a further round of screening. In addition, research in the United Kingdom has consistently shown low public awareness of the early warning signs of breast cancer (Brunswick *et al*, 2001; Wardle *et al*, 2001; Grunfeld *et al*, 2002; Adlard and Hume, 2003; Moser *et al*, 2007) particularly among older women (Linsell *et al*, 2008). This is thought to be more likely to explain survival differences between the United Kingdom and other countries than differences in availability or uptake of breast screening (Richards, 2009). Another reason to evaluate interventions to promote breast cancer awareness is that it may have implications for increasing cancer awareness and survival in other cancers, for which screening programmes may not be available.

We have shown that our intervention, a 10-min interaction with a health professional plus a booklet, promotes breast cancer awareness in older women after 12 months. This trial was not designed to show whether it will promote early presentation in breast cancer and thereby improve survival, although we do plan to evaluate the effect on screening uptake 3 years after randomisation. There is an established association between delayed presentation and survival (Richards *et al*, 1999), but the evidence linking cancer awareness and early presentation is less strong. There is evidence that poor knowledge of non-lump symptoms is associated with delay in presentation (Ramirez *et al*, 1999). Other evidence is indirect: women belonging to populations most likely to have delayed diagnosis of breast cancer (Ramirez *et al*, 1999), also have lower cancer awareness, including older women, women of lower socio-economic status and black women (Grunfeld *et al*, 2002; Scanlon and Wood, 2005; Linsell *et al*, 2008). We acknowledge that this trial does not provide evidence that our intervention will promote early presentation, although that is its ultimate aim. We plan further trials to examine whether the intervention reduces delay in presentation, but these, much larger, studies would not be possible without first building the evidence for its effect on breast cancer awareness, which we hypothesise is on the causal pathway.

Delay in presentation in breast cancer is an important public health problem. We estimate that 7000–12 000 women delay presentation for >3 months in England each year (Richards *et al*, 1999; Office for National Statistics, 2008). Women who delay presenting for 3 to 6 months have 7% lower 5-year survival than those with shorter delays (Richards *et al*, 1999). If only 7000 women per year in England delay presentation for >3 months, about 500 will die as a result (assuming a 5-year breast cancer survival of 80% in women who delay <3 months and 73% in those who delay >3 months). If we find that the intervention reduces breast cancer mortality, it could be one of the key elements of a programme to bring UK breast cancer survival closer to the standards obtained in similar countries. It may also deliver other benefits, to women themselves and the NHS, as a result of less intensive breast cancer treatment given at an earlier stage.

## Conflict of interest

The authors declare no conflict of interest.

## Figures and Tables

**Table 1:** Baseline characteristics of participants

	**Usual care (*n*=287)**	**Booklet (*n*=292)**	**Interaction plus booklet (*n*=284)**
*Relationship status, n* (%)	(*n*=285)	(*n*=283)	(*n*=282)
Married or cohabiting	162 (56.8)	151 (53.4)	174 (61.7)
Widowed	56 (19.7)	61 (21.6)	50 (17.7)
Single	22 (7.7)	28 (9.9)	17 (6.0)
Divorced or separated	45 (15.8)	43 (15.2)	41 (14.5)
			
*Education, n* (%)	(*n*=266)	(*n*=269)	(*n*=263)
No formal qualifications	93 (35.0)	109 (40.5)	116 (44.1)
O level or school certificate	89 (33.5)	81 (30.1)	77 (29.3)
A level or higher school certificate	34 (12.8)	31 (11.5)	29 (11.0)
Degree or above	50 (18.8)	48 (17.8)	41 (15.6)
			
*Ethnic group, n* (%)	(*n*=281)	(*n*=284)	(*n*=280)
White British	187 (66.6)	196 (69.0)	186 (66.4)
White other	23 (8.2)	23 (8.1)	32 (11.4)
Black-Caribbean	36 (12.8)	38 (13.4)	37 (13.2)
Other	35 (12.5)	27 (9.5)	25 (8.9)
			
*Index of multiple deprivation, median rank (IQR)*	(*n*=286)	(*n*=292)	(*n*=284)
(1 (most) to 32 482 (least) deprived)	14 557 (8222–5989)	16 511 (8809–6184)	15 375 (8575–5729)

**Table 2:** Breast cancer awareness and component items at 1 month and 1 year after randomisation

	**Baseline**			**1 month**			**1 year**		
	**Usual care**	**Booklet**	**Interaction plus booklet**	**Usual care**	**Booklet**	**Interaction plus booklet**	**Usual care**	**Booklet**	**Interaction plus booklet**
*Breast cancer awareness*									
Number (%) breast cancer aware^a^	9/267 (3.4)	8/275 (2.9)	5/272 (1.8)	10/244 (4.1)	30/237 (12.7)	75/229 (32.8)	9/229 (3.9)	26/227 (11.5)	53/225 (23.6)
Odds ratio (95% CI), *P*-value (*vs* usual care)				1.0	4.4 (1.6–2.0) *P*=0.004	24.0 (7.7–73.7) *P*<0.001	1.0	3.5 (1.2–10.5) *P*=0.025	15.2 (4.8–47.8) *P*<0.001
									
*Knowledge of breast cancer symptoms*									
Number (%) identifying ⩾5 non-lump symptoms	111/284 (39.1)	126/286 (44.1)	122/280 (43.6)	136/251 (54.2)	151/245 (61.6)	187/237 (78.9)	142/233 (60.9)	167/236 (70.8)	170/230 (73.9)
Odds ratio (95% CI), *P*-value (*vs* usual care)				1.0	1.1 (0.8–1.5) *P*=0.61	2.5 (1.7–3.6) *P*<0.001	1.0	1.3 (0.9–1.9) *P*=0.23	1.7 (1.1–2.4) *P*=0.01
									
*Knowledge of age-related risk*									
Number (%) identifying a 70-year-old woman as most likely to get breast cancer	30/269 (11.2)	36/282 (12.8)	28/276 (10.1)	22/254 (8.7)	62/249 (24.9)	109/244 (44.7)	16/234 (6.8)	53/237 (22.4)	78/234 (33.3)
Odds ratio (95% CI), *P*-value (*vs* usual care)				1.0	3.2 (1.8–5.8) *P*<0.001	9.5 (5.1–17.6) *P*<0.001	1.0	3.4 (1.8–6.7) *P*<0.001	7.4 (3.7–14.7) *P*<0.001
									
*Breast checking*									
Number (%) reporting breast checking at least once a month	152/285 (53.3)	139/288 (48.3)	154/284 (54.2)	163/261 (62.5)	157/256 (61.3)	192/247 (77.7)	171/239 (71.6)	169/243 (70.0)	180/234 (76.9)
Odds ratio (95% CI), *P*-value (*vs* usual care)				1.0	1.2 (0.9–1.6) *P*=0.25	2.0 (1.4–2.8) *P*<0.001	1.0	1.1 (0.8–1.6) *P*=0.47	1.3 (0.9–1.8) *P*=0.23

a A woman scored three points on the breast cancer awareness score if she: identified at least five non-lump symptoms (one point), identified that a 70-year-old woman is most at risk of breast cancer (one point) and reported checking her breasts at least once a month (one point).
